# Choice of time horizon critical in estimating costs and effects of changes to HIV programmes

**DOI:** 10.1371/journal.pone.0196480

**Published:** 2018-05-16

**Authors:** Nicky McCreesh, Ioannis Andrianakis, Rebecca N. Nsubuga, Mark Strong, Ian Vernon, Trevelyan J. McKinley, Jeremy E. Oakley, Michael Goldstein, Richard Hayes, Richard G. White

**Affiliations:** 1 London School of Hygiene and Tropical Medicine, London, United Kingdom; 2 MRC/UVRI Research Unit on AIDS, Entebbe, Uganda; 3 Sheffield University, Sheffield, United Kingdom; 4 Durham University, Durham, United Kingdom; 5 Exeter University, Exeter, United Kingdom; NYU School of Medicine, UNITED STATES

## Abstract

**Background:**

Uganda changed its antiretroviral therapy guidelines in 2014, increasing the CD4 threshold for antiretroviral therapy initiation from 350 cells/μl to 500 cells/μl. We investigate what effect this change in policy is likely to have on HIV incidence, morbidity, and programme costs, and estimate the cost-effectiveness of the change over different time horizons.

**Methods:**

We used a complex individual-based model of HIV transmission and antiretroviral therapy scale-up in Uganda. 100 model fits were generated by fitting the model to 51 demographic, sexual behaviour, and epidemiological calibration targets, varying 96 input parameters, using history matching with model emulation. An additional 19 cost and disability weight parameters were varied during the analysis of the model results. For each model fit, the model was run to 2030, with and without the change in threshold to 500 cells/μl.

**Results:**

The change in threshold led to a 9.7% (90% plausible range: 4.3%-15.0%) reduction in incidence in 2030, and averted 278,944 (118,452–502,790) DALYs, at a total cost of $28M (-$142M to +$195M). The cost per disability adjusted life year (DALY) averted fell over time, from $3238 (-$125 to +$29,969) in 2014 to $100 (-$499 to +$785) in 2030. The change in threshold was cost-effective (cost <3×Uganda’s per capita GDP per DALY averted) by 2018, and highly cost-effective (cost <Uganda’s per capita GDP per DALY averted) by 2022, for more than 50% of parameter sets.

**Conclusions:**

Model results suggest that the change in threshold is unlikely to have been cost-effective to date, but is likely to be highly cost-effective in Uganda by 2030. The time horizon needs to be chosen carefully when projecting intervention effects. Large amounts of uncertainty in our results demonstrates the need to comprehensively incorporate uncertainties in model parameterisation.

## Introduction

The World Health Organization (WHO) published its first guidelines for antiretroviral therapy (ART) provision in resource limited settings in 2002, at which time it recommended that ART be provided for all people living with HIV with CD4 counts of <200 cells/μl[1]. Since then, WHO’s recommended threshold for initiating ART has increased over time, reaching <500 cells/μl in 2013[2]. From September 2015, WHO has recommended universal access to ART for all people living with HIV[3].

ART first became freely available through the Ministry of Health in Uganda in 2003, with local guidelines recommending ART initiation at CD4 counts of <200 cells/μl[4]. This threshold was increased gradually over time, to 250 cells/μl in 2009, 350 cells/μl in 2010, and 500 cells/μl in 2014[5].

Mathematical modelling provides one way of estimating the costs and effects of changes in ART guidelines. Previous studies have investigated the cost-effectiveness of changes in guidelines in a number of different sub-Saharan African countries and settings, including South Africa[6–8], Eastern Africa[9], and Zambia[10]. In this study, we use mathematical modelling to investigate what effect Uganda’s 2014 change in policy (from ART at CD4 counts <350 cells/μl to <500 cells/μl) is likely to have on HIV incidence, morbidity, and mortality. We also estimate programme costs, and the cost-effectiveness of the change over time. We provide a comprehensive measure of the level of uncertainty in all our results.

## Methods

### Model description

This study was conducted using an individual-based model of HIV transmission and care, described in full in S1 and S2 Appendices, reproduced from McCreesh *et al* 2016[11]. The model simulates population demography (births, deaths, and population growth), sexual behaviour (the formation and dissolution of monogamous and concurrent sexual partnerships), HIV transmission, and HIV care. Simulated HIV positive people can be not in care, in pre-ART care, on ART, or dropped out of ART. Movement into care (pre-ART care or on ART) occurs following a positive HIV test and successful linkage to care. People can start ART from pre-ART care or directly following a positive HIV test if they have a CD4 test that indicates that they are below the threshold for ART initiation, if they experience severe morbidity, or if they are pregnant and an Option B+ policy is in place. Receiving ART in the model reduces and individual’s mortality rates, and the probability that they will transmit HIV to their sexual partners.

### ART scale-up and coverage

The model introduces ART in 2003, the year when ART first became freely available in Uganda through Ministry of Health programs[4]. Changes to ART eligibility criteria between 2003 and 2014 were simulated in the model. From 2003–2008, ART was available only to people with CD4 counts below 200 cells/μl, or with WHO stage 3 or 4 conditions. The CD4 threshold for ART initiation increased progressively over time, to 250 cells/μl in 2009, 350 cells/μl in 2010, and to 500 cells/μl in 2014[5]. In addition to this, Option B+, which makes all pregnant women eligible for lifetime ART, was adopted throughout the country by the start of 2014. The model immediately fully implements the change in threshold from 200 to 250 cells/μl at the start of 2009. The other changes in threshold were implemented more slowly in the model, with a proportion of people assumed to seek/obtain treatment at a clinic where the new guidelines were immediately implemented, and the remaining people seeking treatment at a clinic where the guidelines were adopted after a delay of two years. The proportion of people seeking treatments at clinics that immediately adopted new guidelines was controlled by an input parameter that was allowed to vary during model fitting. The plausible range for this parameter was set to 0–1. Option B+ was fully implemented in the model from the start of 2014.

In addition to changes in the ART eligibility criteria, a number of step changes in model parameter values were simulated in various model years. These reflected increases in access to treatment in Uganda, and were necessary to allow the model to fit the empirical ART coverage and initiation data. Step changes in HIV testing rates were modelled in 2005, 2007, and 2012, to allow the model to fit to data on HIV testing coverage over time. Additional step changes in model parameter values in 2008 and 2012 allowed the probability of linking to care following a positive HIV test, the probability of immediately starting ART after testing positive when below the CD4 threshold, and the probability of starting ART following a stage 3 or 4 clinical event to increase over time.

### Model fitting

The model was fitted to routinely collected, countrywide data on the proportion of HIV positive adults (aged 15–49 years) receiving ART in 2005, 2007, 2009, 2011, and 2013, and the proportion of people newly starting ART with a CD4 count of less than 250 cells/μl in the same years[12, 13]. The model was also fitted to data on the proportion of people newly starting ART in 2010 who were women, and the increase in this proportion between 2010 and 2014[12, 14], to capture the effects of the introduction of Option B+. Other fitted outputs included:

- Overall adult (15–49 year old) HIV prevalence in 1991, and adult HIV prevalence by gender in 2004 and 2011[15].
- Rates of dropping out of and restarting ART[16], and 12-month retention on ART[12].
- The proportion of people receiving ART who were on 2^nd^ line treatment in 2010 and 2014[12, 14].
- The proportion of men and women who had ever been tested for HIV in 2004 and 2006, and the proportion of HIV- and HIV+ men and women who had ever been tested for HIV in 2011[17].
- The estimated adult (15–49 year-old) male and female population size in Uganda in 2015, and the growth in population size between 1950–2015[18].
- The incidence and prevalence of monogamous and concurrent sexual partnerships in 2015, based on data from a rural population cohort in South-West Uganda[19–21].

In total, 51 outputs were fitted, and 96 inputs were allowed to vary during the fitting process, incorporating a large number of the potential sources of uncertainty in the correct values of model parameters and output targets. These included the effects of ART on mortality and on HIV transmission. The model was calibrated using history matching with model emulation, which iteratively rejects areas of space where model fits are unlikely to be found[22, 23]. Using this approach, we generated a total of 100 model fits (input parameter combinations) which were consistent with empirical data. Full details of the fitting method are given in McCreesh *et al*[11, 24] and Andrianakis *et al*[25].

### Model scenarios

Two scenarios were simulated. In the first, we simulated ART scale-up in Uganda as it occurred, including the change in guidelines in 2014 which increased the CD4 threshold at which people became eligible for ART from 350 cells/μl to 500 cells/μl. For the second, we simulated a scenario where Uganda did not adopt a CD4 threshold for ART initiation of 500 cells/μl in 2014 and instead retained the 350 cells/μl threshold from 2010.

The model was run for each of the 100 model fits for both scenarios. As the model is stochastic, results were averaged for multiple repetitions (2000) for each fit and scenario.

### Costs and disability adjusted life years (DALYs) averted

Fifteen cost parameters were used to calculate the overall costs to the healthcare system in each scenario. These included programme costs for pre-ART and ART care, 1^st^ and 2^nd^ line drug costs, HIV and CD4 test costs, and healthcare costs arising from HIV-associated morbidity. Costs were considered uncertain, and published data sources were used to determine a plausible range for each cost parameter. Costs are in 2015 USD. For full details, see McCreesh *et al*[11].

Four DALY parameters were used to estimate the effects of adopting the higher CD4 threshold on DALYs averted. These parameters determined the relationship between CD4 count and morbidity, the reduction in morbidity while in pre-ART care, the reduction in morbidity during the first six months on ART, and the disability weight while on established ART. Plausible ranges for disability weights were based on 95% confidence intervals from the Global Burden of Disease Study 2010[26], and data on reductions in rates of hospitalisations after starting cotrimoxazole prophylaxis[27]. DALYs were not age-weighted. Full details are given in McCreesh *et al*[11].

Latin hypercube sampling was used to select 2000 sets of values for the cost and DALY parameters, sampling uniform distributions over their plausible ranges. These were combined with the 100 model fits to obtain 2000 parameter sets, with each model fit being combined with 20 different cost/DALY sets. For each parameter set, the additional costs and DALYs averted that resulted from implementing the higher CD4 threshold were calculated. The net monetary benefit (NMB) of the threshold change was also calculated for each parameter set for a wide range of different values of willingness to pay per DALY averted (WTP, $0-$2500), using the formula *NMB = DALYs averted x WTP–cost*. All costs and DALYs were discounted by 3% per year in the main analysis. In addition, a sensitivity analysis was conducted to explore the effect of the choice of discount rates.

## Results

### Fit to data

**Fig 1:**
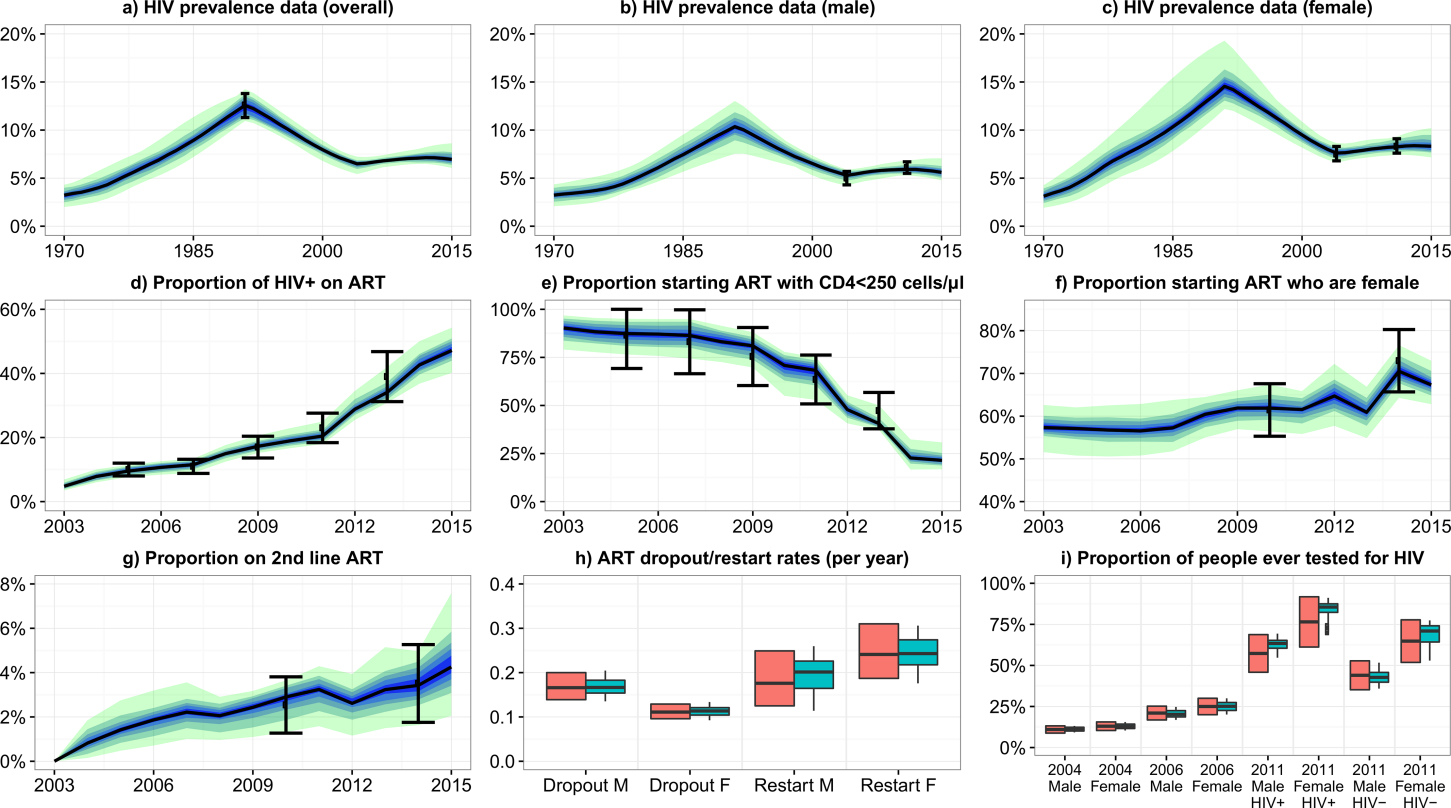
Model baseline fit to empirical data. Graphs a-g: Black dots show the empirical estimates, and the error bars show the plausible ranges for the output values. Black lines show the median model output. Blue/green bands show 10% quantiles of model outputs, from the 100 model fits. The full width of the band shows the range of the model output. Graphs h-i: Orange boxes show the empirical data and plausible ranges. Green boxes show the model output. Model fits to the remaining 20 outcomes are show in McCreesh et al[11].

The model fitted closely to the plausible ranges for all 51 outputs. Fig 1 shows the model fit to the key ART scale-up outputs, as well as the HIV prevalence over time, and ART dropout and restart rates. Fits to the remaining outputs are given in McCreesh *et al*[11].

### Costs, benefits, and cost-effectiveness

**Fig 2:**
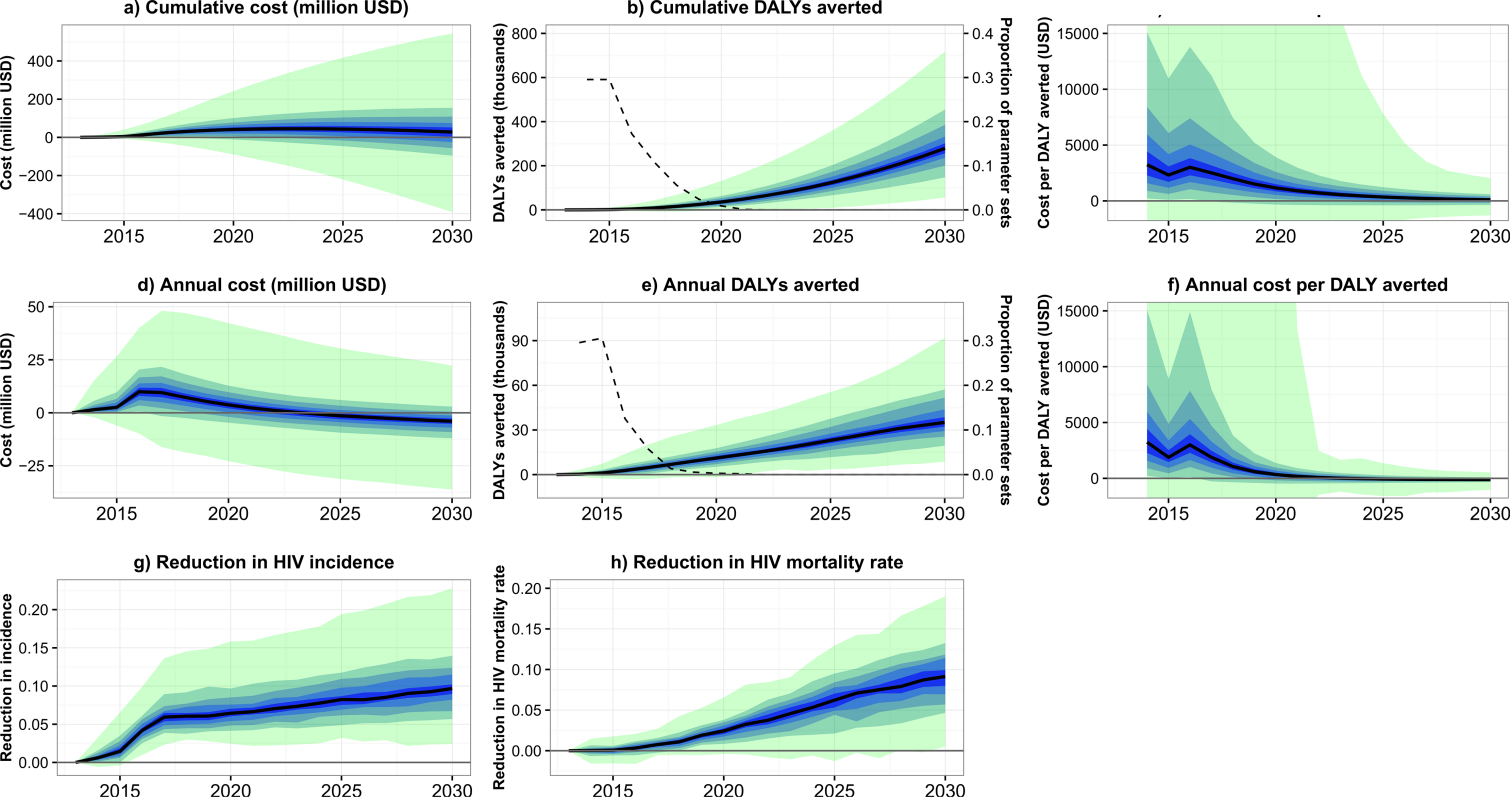
Costs and effects over time of the change in CD4 threshold. a) Total additional costs over time (cumulative). b) Total DALYs averted over time (cumulative) (bands) and proportion of parameter sets where the number of DALYS averted was negative (dashed line, second axis). c) Total cost per DALY averted over time (parameter sets are excluded if the cumulative number of DALYs averted by that year are negative). d) Annual additional costs over time. e) Annual DALYs averted over time (cumulative) (bands) and proportion of parameter sets where the number of DALYS averted was negative (dashed line, second axis). f) Annual cost per DALY averted over time (parameter sets are excluded if the number of DALYs averted in that year are negative). g) Reduction in annual HIV incidence with the change in CD4 threshold, compared to scenario with no change. h) Reduction in annual HIV mortality rates with the change in CD4 threshold, compared to scenario with no change. Black lines show the median model output, and blue/green bands show 10% quantiles of model outputs, from the 2000 parameter sets.

In the model, increasing the CD4 threshold for ART initiation increased annual costs by a maximum of $10 million (90% plausible range: -$89,526 to +$26 million) in 2016 (2014–2017) (Fig 2D). Cumulative costs increased over time to a maximum of 47 million USD (90% plausible range: -$89,526 to +$196 million) in 2023 (2014–2030), before falling to 28 million USD (-$142 million +$195 million) in 2030 (Fig 2A). The change in threshold was cost saving by 2030 in 39% of parameter sets.

Increasing the CD4 threshold for ART initiation averted a total of 278,944 (90% plausible range: 118,452–502,790) DALYs by 2030 (Fig 2B). In contrast to the reductions in HIV incidence, the rate at which DALYs were averted increased over time, with over half the DALYs averted being averted during the five years from 2026 to 2030, and the highest number of DALYs being averted in 2030 (35,084 (15,129 to 66,965), Fig 2E). The very small effect of the change in threshold on DALYs averted in the years immediately following the introduction of the new threshold, combined with the stochastic nature of the model, meant that for some parameter sets the overall number of DALYs averted was negative during the first few years of the intervention. This fell rapidly from 31% of parameter sets in 2014, to <1% by 2019.

**Fig 3:**
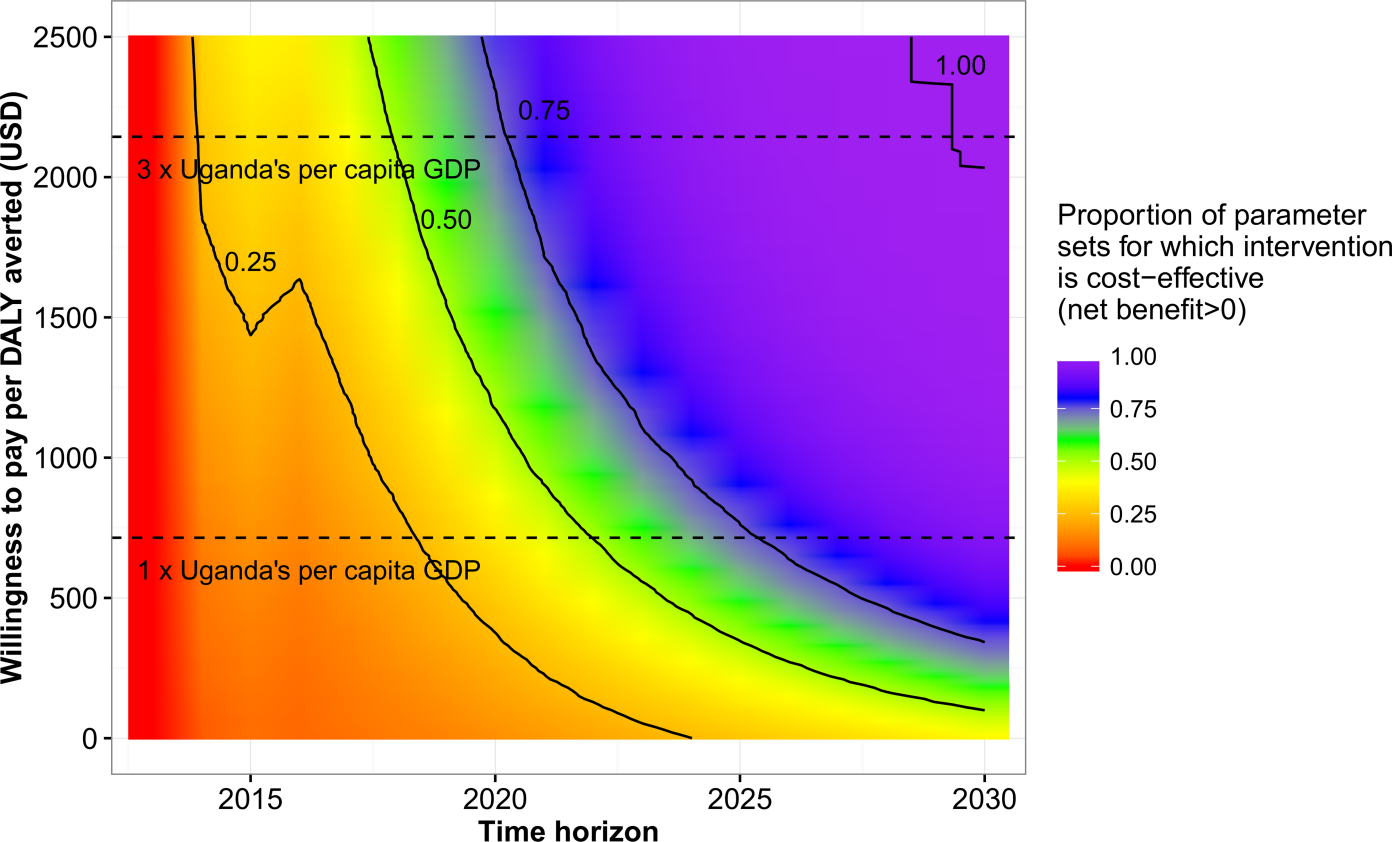
Probability that the change in CD4 threshold is cost-effective, by time horizon and willingness to pay per DALY averted. Black lines indicate where 25%, 50%, 75%, and 100% of parameter sets are cost-effective. Horizontal dashed lines indicate one and three times Uganda’s per capita GDP.

The total cost per DALY averted fell over time, from a maximum of $3238 (90% plausible range: -$125 to +$29,969) during the first year after the introduction of the change in threshold, to a minimum of $100 (-$499 to +$785) in 2030 (Fig 2C). The annual cost per DALY averted fell from $3238 (-$125 to +$29,969) in 2014 to -$114 (-$408 to +$159) in 2030 (Fig 2F). The cost per DALY averted increased slightly between 2015 and 2016, as the change in threshold was assumed to be fully implemented in all clinics in 2016. Fig 3 shows the probability that the change in threshold was cost-effective, by year and willingness to pay per DALY averted (WTP). During the first year after implementation, it was highly unlikely that the intervention was cost-effective (had a positive net benefit), even at a high WTP of $2500 per DALY averted. By 2030, the intervention was cost-effective for more than 50% of parameter sets at a WTP of $100, 14% of Uganda’s per capita GDP. The World Health Organization (WHO) considers interventions to be cost-effective if they cost less than three times a country’s per capita GDP per DALY averted, and highly cost-effective if they cost less than one times a country’s per capita GDP per DALY averted. Using these WTP values, for more than 50% of parameter sets, the change in threshold was cost-effective by 2018, and highly cost-effective by 2022.

Our results were relatively insensitive to the choice of discount rates, with the cost per DALY averted in 2030 ranging from $62 ($-487 to +$666) with no discounting, to $143 ($-513 to +$901) with costs and DALYs discounted by 6% per year (Table 1). Using WHO criteria, with all discount rates we considered, the intervention first became cost-effective in 2018, and highly cost-effective in 2022–2023.

**Table 1:** Effect of choice of cost and DALY discount rates on intervention cost-effectiveness.

Discount rates (per year)			Year in which, for >50% of parameter sets, the intervention first becomes:	
Costs	DALYs	Cost per DALY averted in 2030 (90% CI)	Cost effective (cost/DALY averted <3×Uganda per capita GDP)	Highly cost-effective (cost/DALY averted <1×Uganda per capita GDP)
0.0%	0.0%	61 (-487 to 666)	2018	2022
1.5%	1.5%	80 (-491 to 723)	2018	2022
3.0%	3.0%	100 (-499 to 786)	2018	2022
6.0%	6.0%	143 (-513 to 901)	2018	2023
3.0%	1.5%	87 (-432 to 683)	2018	2022
6.0%	1.5%	128 (-649 to 1012)	2018	2022

Compared to a scenario where Uganda did not adopt the CD4 500 cells/μl threshold, adopting the threshold led to a 9.7% (90% plausible range: 4.3%-15.0%) reduction in incidence in 2030 (Fig 1G). Much of the reduction in incidence occurred during the first 4 years after the change in guidelines, with a 5.9% (3.5%-9.7%) reduction in incidence by the end of 2017. Adopting the threshold led to a gradual reduction in the HIV mortality rate over time (compared to the scenario where the change in threshold was not adopted), up to a maximum of 9.1% (3.4%-14%) in 2030 (Fig 1H).

## Discussion

Model results suggest that the change in ART eligibility criteria made by Uganda in 2014—increasing the CD4 threshold to 500 cells/μl—is highly unlikely to have been cost-effective during the first few years following the change in guidelines, with an estimated cost per DALY averted of $2715 (90% plausible range: +$219 to +$15,106) in 2014. Cost-effectiveness will increase over time however, and by 2030 we estimate that the change in guidelines will have had an overall cost of $100 per DALY averted (-$365 to +$593). The increase in cost-effectiveness over time occurred both through increases in the rate at which DALYs were averted, and falls in the cost of the intervention over time.

Our study highlights the critical importance to the results of mathematical modelling studies of two key types of assumptions or choice. The first is time horizon over which interventions are simulated. Using WHO thresholds for cost-effectiveness, with time horizons of six years of less, six to nine years, and ten or more years, the intervention we consider here would be deemed not cost-effective, cost-effective, and highly cost-effective respectively. This reflects the fact that the costs of the intervention are initially high, before falling in later years, while the number of DALYs averted each year increases over time. The choice of time horizon is likely to be similarly important when evaluating the costs and effects of most HIV interventions or programmes, due to the long durations of HIV infections, and increasing morbidity and mortality with increasing time since infection.

The second is assumptions made during model development and parameterisation. In this study, we comprehensively incorporate large amounts of the uncertainty that exists in model inputs and fitted outputs, by calibrating the model using history matching with model emulation. Additional uncertainty in costs and disability weights was also incorporated during the analysis of the model output. Providing realistic estimates of uncertainty is vital to allow policy makers to make informed decisions. It is often neglected in mathematical modelling studies however, which frequently provide only point estimates, or the results of limited sensitivity analyses. This study shows that when uncertainty in current conditions is comprehensively incorporated, the uncertainty in results can be very large. Based on our analysis, the 90% plausible range in 2030 for number of DALYS averted by the change in CD4 threshold was 118 to 503 thousand, for total cost -$89,526 to +$196 million, and for cost per DALY averted was -$499 to +$785.

A limitation of our study is that we do not incorporate any changes to ART policy, coverage of male circumcision or other interventions, or changes in population sexual behaviour, that occur in Uganda after 2015. If changes occur that result in a lower incidence of HIV infection, then our results are likely to overestimate the costs, DALYs averted, and cost-effectiveness of the intervention. If changes result in a higher HIV incidence, then the cost, effects, and cost-effectiveness of the intervention are likely to be underestimated. Changes to HIV care policy and/or implementation, or the effectiveness of ART (e.g. improved regimens or increased drug resistance) will have more variable and unpredictable effects on the costs, benefits, and cost-effectiveness of the change in policy.

## Conclusions

Our model results suggest that the 2014 change in CD4 threshold in Uganda from 350 cells/μl to 500 cells/μl is unlikely to have been cost-effective to date, but is likely to be highly cost-effective by 2030. When projecting intervention effects, both the choice of time horizon and a comprehensive approach to incorporating uncertainty can have a large effect on results and conclusions.

## Supporting information

S1 Appendix Technical model description. (DOCX)

S2 Appendix Model and data description. (DOCX)
