# Supplementary material for: Choice of time horizon critical in estimating costs and effects of changes to HIV programmes
Source: PLoS One. 2018 May 16;13(5):e0196480. doi: 10.1371/journal.pone.0196480 (PMC5955498; doi:10.1371/journal.pone.0196480)
Supplement: S2 Appendix — (DOCX) [file pone.0196480.s002.docx]

# Model and data description

## Demography

People entered into the model at age 15, and were removed from the model at age 50. Between these two ages, constant male and female non-HIV mortality rates were simulated. Mortality rates were set equal to the estimated mortality rates for men and women aged 15-49 in Uganda in 1975-1980[1]. Mortality rates for these years were used as they were the years when adult mortality rates were lowest in Uganda, before HIV greatly increased adult mortality rates (HIV mortality is simulated separately in the model). A constant birth rate per simulated women alive 15 years earlier was modelled. A wide plausible range around birth rates was used, to allow the model to be fitted to demographic outputs.

The model was fitted to the estimated 15-49 year old male and female population size in Uganda in 2015[1], and to the projected growth in the number of adult men and women in Uganda between 1950-2015. To reduce the run time of the model, one model person was simulated for each 2000 people in Uganda.

## Sexual behaviour

Due to a lack of detailed country-wide information on sexual behaviour, the model was parameterised using and fitted to data on sexual behaviour from the Masaka General Population Cohort in rural south-west Uganda (GPC)[2]. Two sexual behaviour risk groups were modelled (high and low risk), and two partnership concurrency groups (high and low concurrency). On entry into the model, each person was assigned to a risk group (with a probability that varied according to their gender), and to a concurrency group (with a probability that varied according to their gender and risk group).

Data from 2195 men and 3022 women from the GPC in 2009/10 were used to determine the proportion of men and women in the high risk group, and the sexual behaviour of men and women in the high and low risk groups. Individuals were classed as high risk if they reported more than the 75^th^ percentile number of lifetime sexual partners for their age (in 5 year age groups) and gender. This was then age standardised in 5 year age groups to the age and gender distribution of adults aged 15-49 years in Uganda as a whole in 2015[1], and resulted in 21% of men and 15% of women being classed as high risk. Data on reported number of ongoing partnerships were used to estimate the age-standardised proportions of high and low risk men and women with 0, 1, and 2+ ongoing partnerships. The model (in 2015) was fitted to the proportion of high and low risk men with 0, 1, and 2+ ongoing partnerships, and the proportion of high and low risk women with 0 ongoing partnerships. As few women reported more than one ongoing partnership[3], low risk women were assumed not to form concurrent partnerships. As the total number of partnerships offered by men in the model had to be equal to the total number offered by women, the model was not fitted to data on the proportion of high risk women reporting 1 or 2+ partnerships.

Data on annual partnership incidence (new partnerships formed per year) were available from a subset of the GPC in 2004-2008. Data on reported partnership incidence in men were age standardised to the age distribution of adults aged 15-49 years in Uganda in 2015. Male partnership incidence in 2015 in the model was fitted to this value. Data on reported male partnership prevalence and incidence were used to determine a plausible range for the model input mean partnership duration.

To allow the model to be fitted to trends in HIV prevalence over time in Uganda, two step changes in sexual behaviour were simulated in 1992 and 2004. These ranges were chosen as HIV prevalence peaked in 1991 in Uganda, and started to rise and again from 2004[4].

## Health seeking behaviour/adherence

People were assigned at entry to a high or low adherence group. Probabilities of being assigned to the low adherence group could be different for men and women, and were higher for people with high risk sexual behaviour than for people with low risk sexual behaviour. Plausible ranges for the proportion of high risk men and women assigned to the high adherence group were considered to be 0.5-0.9 (Technical model description, Table 4). Plausible ranges for the proportion of low risk men and women assigned to the high adherence group were considered to be 0.8-1 (Technical model description, Table 4). Additional constraints were placed on the ranges, such that the proportions of low risk men and women assigned to the high adherence group could not be less than the proportions of high risk men and women assigned to the high adherence group.

The high and low adherence groups were designed to represent a wider range of behaviours than just adherence to prescribed ART drug regimens. People in the model in the low adherence group had lower HIV testing rates, a lower rate of linking to care following a positive HIV test, lower probabilities of starting ART following stage 3 or 4 clinical events when not in care, higher rates of dropping out of pre-ART care and ART, lower rates of restarting ART after dropping out, and higher rates of developing drug resistance. The adherence groups therefore also incorporated differences in health seeking behaviour, access to care, and quality of care available (e.g. frequency of drug stock outs).

Concern has been expressed that increasing the CD4 threshold for starting ART may lead to lower adherence, due primarily to people starting ART without having experienced the negative health consequences of more advanced infections. Evidence for this is mixed however[5-8]. In the model, people starting ART for reasons other than having recently experienced a stage 3 or 4 clinical event could be moved from the high adherence group to the low adherence group. The plausible range for the probability of this occurring was set to 0-0.5 (Technical model description, Table 4).

## HIV natural history

### HIV introduction

HIV was introduced into the model in 1970 by infecting a proportion of high risk people and a proportion of low risk people (chosen at random) with HIV.

### CD4 count

CD4 count was used in the model to determine HIV mortality rates and transmission probabilities. It was modelled as a continuous variable, and tracked over time for each simulated person. Each person was assigned an initial CD4 count and rate of √CD4 count decline at entry into the model. A plausible range for the mean rate of √CD4 count decline when HIV+ was determined using data from Uganda[9]. Mean initial CD4 count in men and women was modelled as a function of the mean rate of √CD4 count decline. The functions were chosen to give plausible CD4 counts at 10 months following infection in men and women, based on data from Uganda [10]. The standard deviation for the initial CD4 count was chosen to give a plausible range of CD4 counts at 10 months following infection.

CD4 counts declined in the model when someone was HIV+, and not on ART. They did not decline when someone was HIV- or on ART. Upon dropping out of ART, a step change (increase or decrease) in CD4 count was simulated. The size and direction of the change could be different for people dropping out after less than or greater than one year continuously on 1^st^ or 2^nd^ line ART. The step changes in CD4 count were designed to reflect both the gradual rise in CD4 count that typically occurs when someone is taking ART, and any drop that may have occurred during the initial three months after dropping out (dropping out was defined in the model as a continuous break from ART of three or more months. See section ‘retention in care’ for more details). A maximum CD4 count on dropping out was used to prevent implausible CD4 counts being reached through a person repeatedly dropping out of and restarting ART. This was very unlikely to occur in the fitted model, but could occur with certain combinations of parameters during model fitting. Large plausible ranges were selected for the simulated changes in CD4 count upon dropping out of ART, as the data that were available to inform parameterisation come from research studies (e.g. [11-14]), and are unlikely to reflect changes in CD4 count in more typical settings among people with poor adherence to treatment.

### Primary infection

Due to higher transmission probabilities during primary infections (see section HIV transmission), the model tracked which HIV+ people had primary infections. People in the model were considered to develop primary infections immediately after infection. Individuals had their own primary stage duration, drawn from a normal distribution. The plausible range for the mean primary stage duration was set to 1-8 months (Technical model description, Table 4)[15]**.**

### Mortality

In addition to a background mortality rate, HIV+ people in the model could die due to HIV related causes. This could occur at any CD4 count, however the HIV mortality rate increased with decreasing CD4 count. Plausible ranges for HIV mortality rates while not in care were taken from a study conducted in Uganda before the widespread availability of ART[16]. Simulated mortality rates for ART drop outs were set equal to mortality rates for people who were not in care and had never been on ART. Simulated mortality rates were reduced for people in pre-ART care. The plausible range for the reduction was determined from data on the effects of cotrimoxazole prophylaxis on mortality rates[16], and coverage of cotrimoxazole prophylaxis among people in pre-ART care in Uganda[17, 18].

Data were available on HIV mortality rates after more than one year on ART in Uganda, stratified by CD4 count at ART initiation[19]. These were used to determine plausible ranges for mortality rates in the model for people in their 2^nd^ and subsequent continuous years on ART. HIV mortality rates for people on ART were assumed to be higher in the 1^st^ year after starting ART than in subsequent years. Mortality rates in the first year on ART were therefore calculated as a weighted average of mortality rates in pre-ART care and on established ART, at the same CD4 count. Mortality rates were also assumed to be higher in the 1^st^ year after switching to 2^nd^ line ART, as the majority of people being switched are likely to have poorly controlled HIV infections at the time of the switch (due to resistance to the 1^st^ line regimen and/or due to low adherence). If selected mortality rates on ART were higher than selected mortality rates in pre-ART care at any CD4 count, then the mortality rates on ART at that CD4 count was set equal to mortality rate in pre-ART care at the same CD4 count.

Mortality rates for individuals on ART can increase due to drug resistance. This is described in the section ‘Effects of resistance on mortality’.

The model was fitted to data from eastern Africa on the median time between infection and death in the absence of ART[20].

## HIV prevalence

The model was fitted to estimates of overall adult (15-49 years) HIV prevalence in Uganda in 1991, and estimates of HIV prevalence in men and women aged 15-49 in 2004 and 2011. 1991 and 2004 were chosen as fitting years for HIV prevalence as they were the years when HIV prevalence was estimated to have peaked in Uganda (1991), and reached a local minimum (2004)[4]. Fitting to these years therefore ensures that the model fits the overall trend in HIV prevalence over time in Uganda. In addition, HIV prevalence data by gender were available from a national prevalence survey in 2004[21]. 2011 was chosen as it is the most recent year for which accurate estimates of HIV prevalence are available from a nationwide survey[22].

## HIV care and ART

### Overview

The model was designed to accurately represent the key features of major routes into and through pre-ART care and ART in Uganda. Figure 1 gives a summary of the simulated care pathway, and further details of routes through the care pathway are given below.


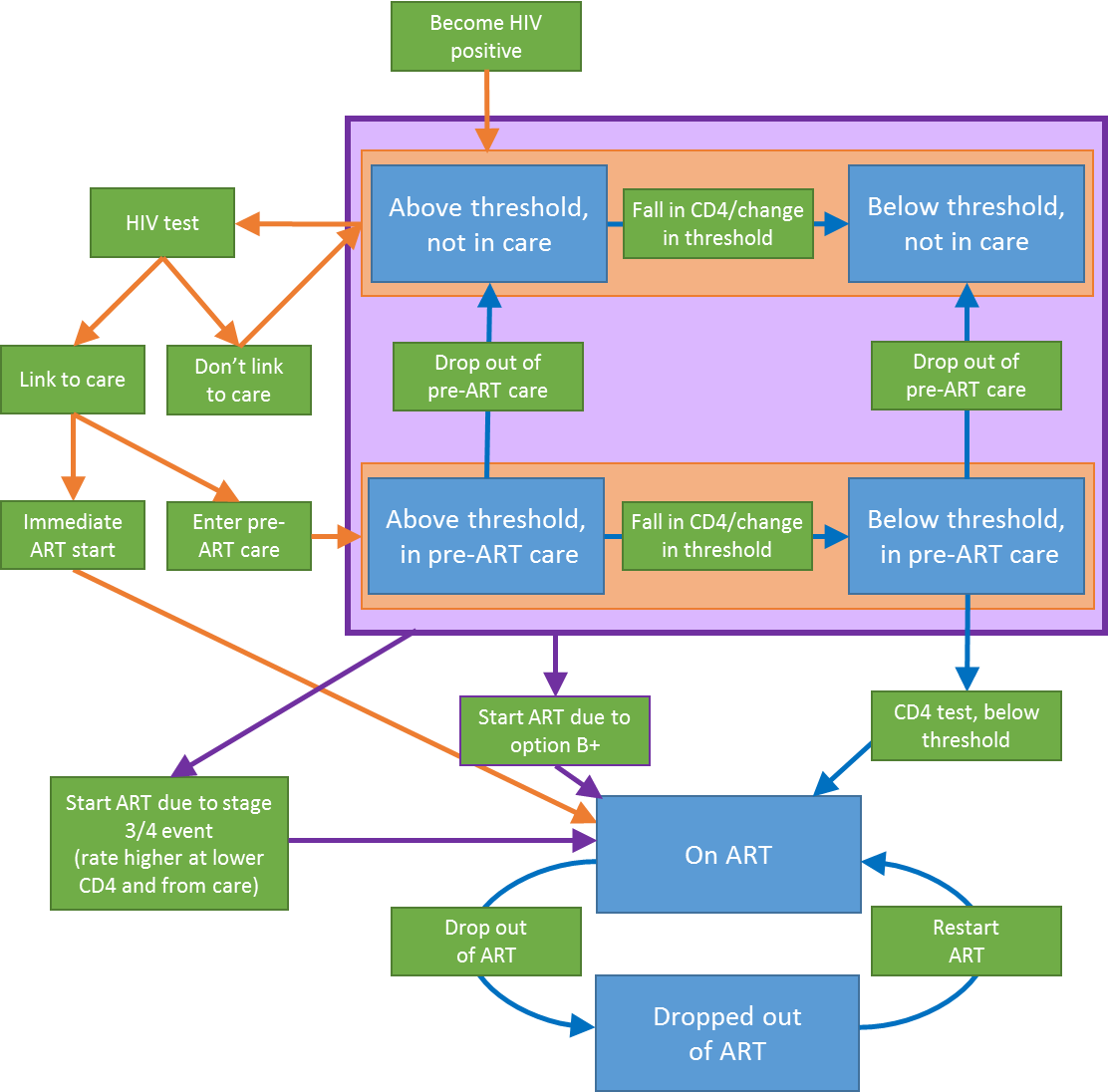


Figure 1. Summary of the simulated care pathway.

### HIV testing

A constant rate of HIV testing was simulated in the model. The rate could be different for men and women, was lower for people in the low adherence group, and could be higher in HIV+ people. People could only test for HIV if at least six months had passed since their last HIV test. If people tested within one month of being infected with HIV, there was a 75% chance that the test would give a false negative result[23]. Under these circumstances, people in the model could test again the following month, and did not have to wait six months before their next test. HIV testing started in 1990 in the model. The model was fitted to data on the overall portion of adult men and women who reported ever having been tested for HIV and received the results in 2004 and 2006, and data on the proportion of HIV- and HIV+ men and women who reported ever having been tested for HIV and received the results in 2011[22].

From 2003 (when cotrimoxazole prophylaxis started to become available in Uganda[24]), upon testing positive, a proportion of men and women could be immediately linked to care. From 2003, a proportion of those linked to care who were below the CD4 threshold for ART initiation could also immediately start ART.

### Pre-ART care

Pre-ART care was explicitly simulated in the model. People in pre-ART care had lower mortality rates than people with similar CD4 counts who were not in care (see section ‘mortality’), and had higher rates of starting ART.

People could drop out of pre-ART care in the model. The rate of dropping out could be different in men and women, and was higher in people with low adherence. Data on retention in pre-ART care in Uganda are limited[25, 26], and mean rates of dropping out of pre-ART in routine settings could not be calculated. It is unlikely, however, that dropout rates from pre-ART are lower than dropout rates from ART. Pre-ART care dropout rates were therefore set to be proportion to ART dropout rates, multiplied by a scaling factor. The plausible range for the scaling factor was set to be 1-2 (Technical model description, Table 4).

Ugandan Ministry of Health reports provide data on the numbers of people in pre-ART care and ART care in 2010, 2011, and 2013[18, 27, 28]. These were used to calculate estimates of the proportion of people in HIV care (pre-ART or on ART) who were on ART in the three years, and the model was fitted to the estimates. Due to potential limitations of the data (which calculated from routine data submitted by health centres), large plausible ranges were placed around the point estimates.

### Starting ART

There were five routes onto ART in the model:

1. People could start ART immediately after testing positive, provided that they were below the CD4 threshold. This route is described in the section ‘HIV testing’
2. People not in care could start ART through experiencing a WHO stage 3 or 4 clinical event, regardless of whether or not they were below the CD4 threshold. The rate at which clinical events were assumed to occur increased exponentially with decreasing CD4 count. Two model parameters controlled the rate of starting of ART. One determined the slope of the exponential relationship between CD4 count and clinical events, and its plausible range was informed by data on the rate of clinical events by CD4 count in Côte d’Ivoire[29]. The second parameter determined the proportion of clinical events which led to a person seeking health care and starting ART.
3. People in care could also start ART through experiencing a WHO stage 3 or 4 clinical event. The shape of the relationship between CD4 count and the rate of clinical events was assumed to be the same in HIV+ people in care as in HIV+ people not in care. A separate parameter determined the proportion of clinical events which led to a person who was in pre-ART care starting ART. The proportion of people who started ART following a clinical event could not be lower for people in pre-ART care as for people not in care.
4. People in care could start ART if they had a CD4 test and their CD4 count was below the threshold for starting ART. One parameter controlled the probability of someone having a CD4 test each month. People could not have a CD4 test within six months of their last CD4 test.
5. Option B+ was implemented throughout Uganda at the start of 2014[30]. This means that all HIV+ pregnant women should be started on ART, and retained on it for life, regardless of their CD4 count. In the model, this was controlled using two parameters. One was the pregnancy rate in women aged 15-49. The plausible range for this rate was taken from the UN Department of Economic and Social Affairs’ low and high fertility estimates of fertility in Uganda in 2015. The second parameter controlled the proportion of HIV+ pregnant women who were started on ART in practice. This was allowed to vary between 0-100%, to allow the model to be fitted to data on the proportion of ART initiators who were women before and after the implementation of option B+ (see section ART outputs).

### ART coverage and initiation outputs

Data on ART coverage and characteristics of ART initiators in 2005-2013 were available from routine data collected by the Ministry of Health in Uganda[17, 18]. The model was fitted to data on the proportion of HIV+ adults who were on ART, and the proportion of ART initiators who started with a CD4 count of less than 250 cells/μl, in 2005, 2007, 2009, 2011, and 2013.

To capture the effects of the introduction of option B+ in Uganda, the model was also fitted to data on the proportion of ART initiators who were women in 2010, and the increase in the proportion who were women between 2010 and 2014[17, 31].

### Retention in care

In the model, ‘dropping out of ART’ was defined as a treatment interruption lasting for three months or longer, and was modelled as a separate stage with its own mortality rates and transmission probabilities. Shorter duration treatment interruptions were not explicitly simulated. Rates of dropping out of ART could be different in men and women, and were higher for people in the low adherence group. As dropping out was defined as a treatment interruption of three months or greater, people could not drop out of 1^st^ line ART during the 1^st^ three months. In addition, no increase in drug resistance upon entering the ‘drop out’ stages was simulated, as this increase would have occurred sometime earlier, and was incorporated into the rate of developing resistance when on ART

People could restart ART after dropping out in the model. Rates of restarting could be different in men and women, and were lower for people in the low adherence group. People restarted ART on the same regimen that they were taking when they dropped out (1^st^ or 2^nd^ line), and on restarting their time on ART was reset to zero (i.e. regardless of time previously spent on ART, they experienced the mortality rates of people on ART for the first year for a period of 12 months).

The model was fitted to Ministry of Health estimates of the proportion of ART initiators who were still on ART at 12 months in Uganda in 2006-2014[17]. No trend towards increasing or decreasing retention was observed, and therefore no change in retention over time was simulated, and the model in 2014 was fitted to the mean retention over the 9 year period.

Data were not available from Uganda on rates of stopping and restarting treatment (12 month retention data includes loss to follow up due to mortality). A study in Cape Town, South Africa, recorded rates of dropping out of restarting ART in men and women, with dropping out defined as a treatment interruption of three months or more[32]. Overall rates of dropping out were found to be consistent with data from Uganda on 12 month retention. The model (in 2015) was therefore also fitted to the data on rates of dropping out of and restarting ART in men and women. Rates of dropping out of ART were found to decrease as time on ART increased, and therefore a higher rate of dropping out during the first year on ART was allowed in the model. The plausible range for the higher rate (relative to the rate in subsequent years), was set to 1-2 (Technical model description, Table 4).

### ART scale up

#### Changes in threshold

Although some people had access to ART in Uganda through self-pay, NGOs, or research cohorts prior to 2003, numbers were very small[33]. ART was therefore introduced into the model in 2003, when ART first became freely available through Ministry of Health programs. Initially, ART was available only to people with CD4 counts below 200 cells/μl, or with WHO stage 3 or 4 conditions. The CD4 cut-off has increased progressively since, to 250 cells/μl in 2009, 350 cells/μl in 2010, and to 500 cells/μl in 2014. In addition, option B+ was adopted throughout the country in 2014. These changes in CD4 threshold were incorporated into the model at the start of the year in which the changes were made. The change from 200-250 cells/μl was immediately implemented fully in the model. The changes from 250-350 cells/μl and from 350-500 cells/μl could be implemented more slowly, reflecting a gradual adoption of the new guidelines. A user defined input parameter controlled the proportion of people of who were assumed to seek/obtain treatment at a clinic where the new thresholds were immediately implemented. This lasted for a period of two years, after which all clinics were assumed to fully adopt the new guidelines.

#### Changes in rates

In additional to changes in the CD4 threshold, step changes were also modelled in HIV testing rates (in 2005, 2007, and 2012); and in linkage to care, the probability of immediately starting ART after testing positive (and if below the CD4 threshold), and the probability of starting ART following a stage 3 or 4 clinical event (in 2008 and 2012). These changes reflect increased access to treatment in Uganda, and were necessary to allow the model to fit the ART coverage and initiation data.

## Drug resistance

### Simulated ART regimens

Two drug regimens were simulated in the model, based on the current recommended 1^st^ and 2^nd^ line regimens in Uganda (Table 1). Switching of individual drugs was not simulated, and everyone in the model received the standard 1^st^ or 2^nd^ line regimen. Everyone started ART on the 1^st^ line regimen.

|  | 1^st^ line | 2^nd^ line |
| --- | --- | --- |
| NNRTIs | Efavirenz (EFZ) |  |
| NRTIs | Lamivudine (3TC)  Tenofovir (TDF) | Lamivudine (3TC)  Zidovudine (AZT) |
| PIs |  | Ritonavir-boosted atazanavir (ATV/r) or ritonavir-boosted lopinavir (LPV/r) |

Table 1. Simulated 1^st^ and 2^nd^ line drug regimens

### Model representation of drug resistance

Four types of drug resistance were simulated in the model: major non-nucleoside reverse-transcriptase inhibitors (NNRTI) resistance mutations, major nucleoside reverse transcriptase inhibitors (NRTI) resistance mutations, major protease inhibitor (PI) resistance mutations, and thymidine analogue mutations (TAMs). Each HIV+ person could have no, medium, or high resistance of each type. Each type of resistance reduced the activity of one or more drug, and resistance of each type could arise when individuals were on a drug that selected for that type of resistance. Figure 2 summarises the different interactions between each drug and resistance type in the model.

An overall number of active drugs was calculated for each person on ART in the model. This was done by summing up the level of activity the person had against HIV due to each of the individual three drugs that they were on. If there was no resistance to a particular type of drug, then a person taking that drug had an unweighted activity against HIV of 1. If there was high resistance, then they had an activity of 0. If there was medium resistance, then they had an activity of *medium_resist_value*. As medium resistance incorporated a range of different mutations with different effects on drug effectiveness, a wide plausible range of 0.25-0.75 was allowed for the value of *medium_resist_value* (Technical model description, Table 4)*.* In the model, resistance to tenofovir could occur through either major NRTI resistance mutations, or through the accumulation of TAMs. As either type of mutation(s) will reduce the activity of the drug, the level of activity against HIV of tenofovir was set equal to the lowest level of activity suggested by an individual’s major NRTI mutations and TAMs.

A model structure that simulates fixed 1^st^ and 2^nd^ line drug regimens, and four types of resistance with three levels each, was chosen for two reasons. On one hand, it contains sufficient complexity to allow the most important drivers of drug resistance to be identified, and to determine areas where a better understanding of resistance development and transmission would allow improved predictions to be made (i.e. cause the greatest reductions in confidence interval size). One the other hand, it is sufficiently simple that data are available to inform parameterisation. The choice of the types of resistance to model, and the interactions between the simulated drugs and the different types of resistance, were informed by data from two Stanford University HIV Drug Resistance Databases[34]. The first gives an indication of which mutations are most likely to arise in patients taking specific drugs and treatment regimens, and the second details current knowledge of the effects of each mutation on the activity of different drugs.

Because different classes of drugs may have different levels of activity against HIV, even in the absence of any drug resistance, the activity of each individual drug was weighted according to its class. In line with previously proposed weighting systems[35, 36], the plausible range for the activity level of NRTI class drugs (relative to NNRTI class drugs) was set to 0.5-1, and the plausible range for the activity level of PI class drugs (relative to NNRTI class drugs) was set to 1-2 (Technical model description, Table 4).

### Seeding of resistance

To reflect the use of self-pay mono- and dual-therapy in Uganda prior to 2003, a proportion of HIV+ people were seeded with medium resistance to NNRTI class drugs in 2003.

### Development of resistance

The baseline rate of resistance development in the model was determined by the parameter *acquire_‌resist_‌rate_‌baseline*. Estimates of rates of acquiring drug resistance mutations vary greatly between different studies[37-39]. Furthermore, when estimating rates of developing drug resistance mutations, it is generally assumed that no resistance mutations have arisen in people who are virally supressed. This is unlikely to be true, as viral re-suppression is not uncommon in people with previously detectable resistance mutations[40, 41]. For these reasons, a wide range of resistance development rates were considered plausible, informed by the extreme upper and lower values of 95% confidence intervals from a range of studies and reviews[37-39].

Data suggest that the rate of developing resistance to ritonavir-boosted PI class drugs may be lower than the rate of developing resistance to NNRTI class drugs[42], and therefore the rate of developing resistance to PI class drugs in the model was set to 5-50% of the baseline rate (Technical model description, Table 4). Being on an ART regimen that includes a ritonavir-boosted PI may also reduce the rate at which people acquire resistance to the other drugs in their regimen[42]. The baseline rate of acquiring resistance was therefore reduced by 25-75% in the model when a person was on 2^nd^ line ART (Technical model description, Table 4).

In the model, upon acquiring resistance of any type, a person’s level of resistance of that type increased from none to medium, or from medium from high. With major NNRTI resistance only, people could immediately increase from no resistance to high resistance. The plausible range for the probability of this occurring was set to 50-100% (Technical model description, Table 4). Immediate jumps to high NNRTI resistance were simulated because some common major NNRTI mutations have a very large effect on drug effectiveness, even in the absence of other mutations[34].

People in the low adherence group had a higher rate of acquiring drug resistance mutations in the model than people in the high adherence group. A range of 1.8-11 times higher was considered plausible (Technical model description, Table 4). This was calculated by comparing rates of drug resistance development in people with 100% and <80% adherence, with adherence measured using pill counts[37].

The rate of acquiring additional drug resistance also increased in the model as the number of active drugs that a person was taking decreased. The increase was modelled as a linear function of the number of active drugs, with an input parameter determining the rate of development of drug resistance with only two active drugs compared to the rate with one active drug. The plausible range for the input parameter was taken from a study that compared the prevalence of acquired drug resistance in people with no baseline drug resistance, with the prevalence in people with baseline drug resistance and a median of two active drugs[37].

### Loss of resistance

In the model, once a person had medium or high level resistance of a particular type, then that resistance could not be lost or revert from high to lower levels. It was assumed, however, that resistance mutations would disappear from the majority strain when a person was not on ART. This process was not explicitly modelled, however its effects on the probability of resistance transmission were incorporated into the model. This is discussed fully in the section ‘transmission of resistance’.

We assumed that resistant viruses would quickly become the majority strain once a person started or restarted an ART regimen that had reduced activity as a result of the drug resistance mutation(s). We therefore did not model a period of full drug activity following ART (re)initiation by people with drug resistance mutations.


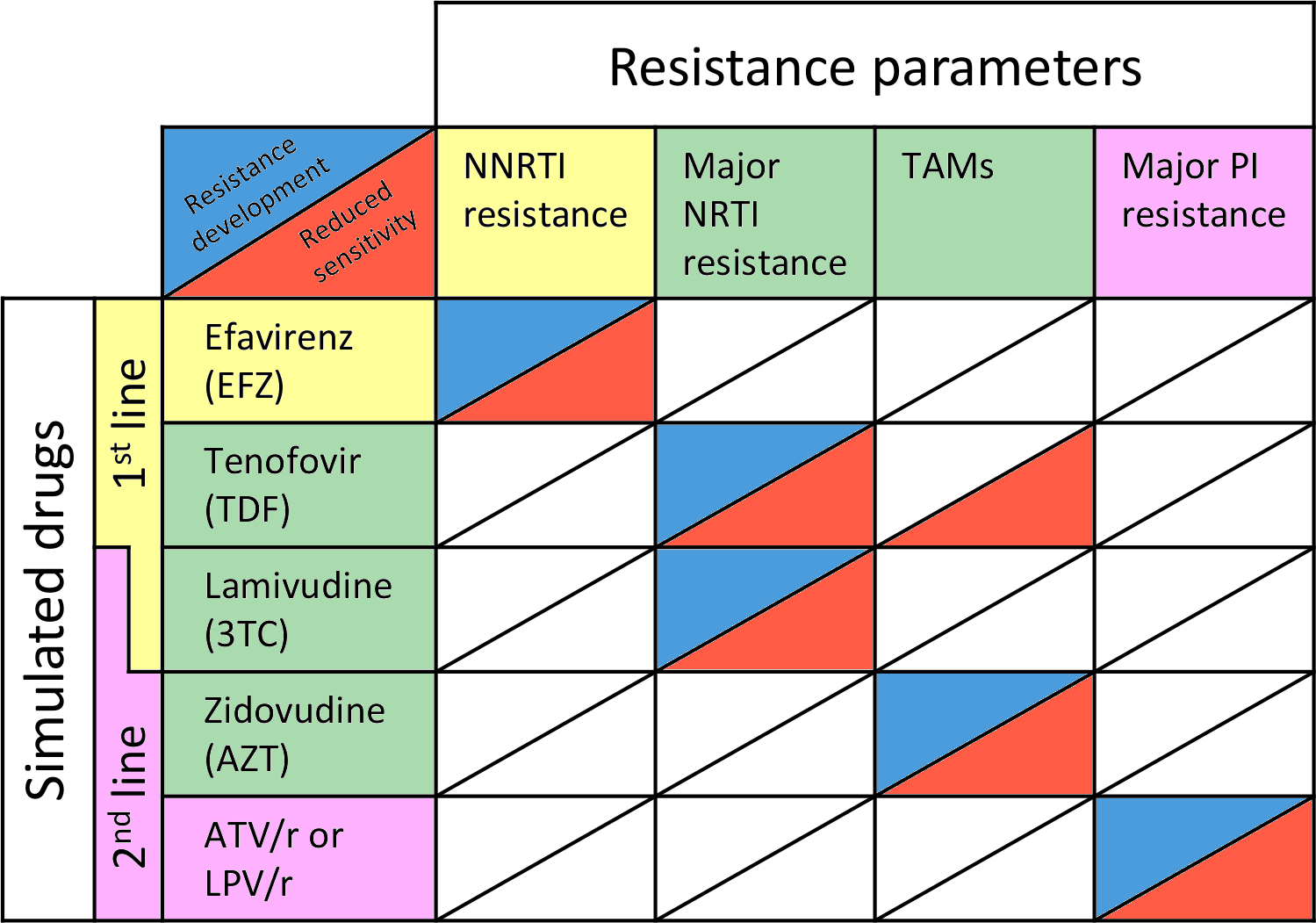


Figure 2. Summary of simulated drugs and model resistance parameters. Yellow indicates NNRTI class drugs, green NRTI class drugs, and pink PI class drugs. Blue indicates that, in the model, the drug could lead to the development of that type of resistance. Red indicates that the resistance parameter led to reduced sensitivity to that drug.

### Effects of resistance on mortality

As people in the model developed resistance to the drug regimen that they on are, their probability of dying each month increased. With the maximum number of active drugs, mortality rates were equal to the rates described in the section ‘mortality’. As the number of active drugs decreased, mortality rates increased exponentially. With zero active drugs, mortality rates on ART were the same as mortality rates for people with similar CD4 counts who were in pre-ART care.

### 2^nd^ line ART

People who had spent more than one year continuously on ART in the model could be switched to 2^nd^ line ART. There were two ways in which this could occur: any person could be switched as a result of drug resistance (‘necessary switching’), and low adherence people could be switched regardless of their level of drug resistance (‘unnecessary switching’).

The rate of necessary switching to 2^nd^ line ART was determined by a single parameter which set the rate at which switching occurred when a person had zero active drugs. With the maximum number of active drugs, a person could not be switched to 2^nd^ line due to drug resistance. Between zero active drugs and the maximum number, the probability of switching declined linearly.

The rate of unnecessary switching was controlled by a single parameter, which gave the probability that each low adherence person (who has been on ART for more than one year continuously) would be switched each month.

The model was fitted to routine data collected by the Ministry of Health on the proportion of adults on ART who were on a 2^nd^ line regimen in 2010 and 2014[17, 31]. Few data were available on what proportion of people are switched to 2^nd^ line ART due to viral failure as a result of drug resistance, and what proportion are switched due to other reasons. Proportions are likely to vary as a result of overall levels of drug resistance in a community, which may increase over time. One study in six African countries in 2007-2009 estimated that, in the absence of access to viral load monitoring, 53% of people who were switched were switched necessarily, where necessarily was defined as a switch occurring when the patient had a viral load of ≥1000 copies/ml[43]. This was used as the central estimate for the proportion of people switched necessarily in the model in 2008. As the definitions of ‘unnecessary switching were different in the study and in the model, a wide range of values from 27-78% were deemed acceptable. (Technical model description, Table 4)

## HIV transmission

### Transmission probabilities

Each month, there was a chance that HIV transmission could occur within discordant partnerships in the model. A coital frequency of ten times per month was simulated, based on data from Uganda[44]. The probability of transmission each month was therefore taken from a binomial distribution with ten trials and a probability of success equal to the transmission probability. There were three components to HIV transmission probabilities in the model. The first was the baseline transmission probability. This was the same for all individuals in the model, and was equal to the input parameter *baseline_transmission*. The second component was determined by the gender of the HIV+ partner. Empirical data suggest that male to female transmission probabilities may be 1.1 to 4.8 times higher than female to male transmission probabilities[45]. This was used as the plausible range for the ratio of male->female to female->male transmission probabilities in the model.

The third component of HIV transmission probabilities was determined by the HIV+ individual’s CD4 count, and whether or not on they were on ART. Transmission probabilities for people with CD4 counts of 200-350 cells/μl (who are not on ART) were used as a baseline in the model, and therefore transmission probabilities for this group were weighted by one. Based on data from a cohort study of discordant couples in seven sub-Saharan African countries, the plausible range for relative transmission probabilites for people with CD4 counts <200 cells/μl and ≥350 cells/μl were considered to be 1.5-6.7 and 0.42-0.95 respectively[46] (Technical model description, Table 4). Transmission probabilities may be much higher for people with primary infections, and the plausible range for relative transmission probabilities with a primary infection was considered to be 1-60[15] (Technical model description, Table 4). An additional joint constraint was placed on the values of the priamary stage transmission probability and primary stage duration, such that they could not both take values at the high end of their plausible ranges (see Technical model description for details). These relative transmission probabilities were used in the model both for people who were ART naïve, and for ART dropouts.

Transmission probabilities for people on ART in the model varied according to their level of drug resistance. With the maximum number of active drugs, the plausible range for relative transmission probabilities was considered to be 0.04-0.21 (Technical model description, Table 4)[47]. As resistance increased, so did transmission probabilities. With no active drugs, transmission probabilities were the same for a person on ART as they were for a person who was not on ART with a similar CD4 count. With intermediate numbers of active drugs, transmission probabilities increased exponentially as the number of active drugs declined.

### Transmission of resistance

Drug resistance mutations could be transmitted in the model. The probability of each mutation being transmitted was independent, and varied depending on whether someone was ART naïve, on ART, or an ART dropout. The plausible ranges for resistance transmission probabilities are shown in Figure 3. For the purposes of transmission, medium level resistance of any type was treated as one mutation, and high level resistance as two. If someone had medium PI resistance, therefore, and the probability of resistance transmission was equal to *p*, a person they infected with HIV would have medium PI resistance with probability *p*, and no PI resistance with probability *1 – p*. If someone had high PI resistance, a person they infected with HIV would have high PI resistance with probability *p^2^*, medium PI resistance with probability *p – p^2^*, and no PI resistance with probability *1 – p*. The probability could be lower for NRTI mutations, as the rate of loss of one very common major NRTI resistance mutation has been shown to be substantially higher than the rate of loss of other mutations[48].

For people in the model who were ART naïve, the probability of resistance transmission was dependent on their CD4 count, and whether they had a primary infection. It is thought that HIV transmission involves the transfer of a single, or very small number, of viruses[49]. If someone is infected with a drug resistant virus, they are therefore unlikely to also be infected with a wild type virus. Losing drug resistance from their majority strain therefore involves a process of back-mutation(s), and is unlikely to occur immediately. For these reasons, the probability of resistance transmission in ART people in the model was highest in people with primary infections, and declined as their CD4 count declined. Plausible ranges for transmission probabilities in each CD4 group (primary, >500 cells/μl, 350-500 cells/μl, 250-350 cells/μl, 200-250 cells/μl, 150-200 cells/μl, 100-150 cells/μl, 50-100 cells/μl, and <50 cells/μl) were calculated from data on the rate of loss over time of (detectable) transmitted drug resistance mutations in ART naïve individuals[49], and data on the rate of decline in CD4 count in HIV+, ART naïve people in Uganda[9].

For people who were on ART in the model, resistance transmission probabilities depended on whether or not they were on an ART regimen that had reduced activity as a result of that particular mutation. If the mutation reduced drug activity, the probability of it being transmitted was assumed to be very high, and the plausible range was set to 0.9-1 (Technical model description, Table 4). If the mutation did not reduce drug activity, transmission probabilities were assumed to be much lower, and the plausible range was set to 0-0.4 (Technical model description, Table 4).

In comparison with people who have been recently infected with HIV, people who have dropped out of ART are far more likely to have wild type viruses in their viral population. This is because they may have been infected with a wild type virus and developed drug resistance mutations during their time on ART, or because back mutations towards the wild type occurred before they started ART. The rate at which they lose drug resistance viruses from their majority strain is therefore likely to occur relatively quickly once the selection pressure is removed. This is supported by empirical data. One study that looked at detectable drug resistance mutations over time in individuals who had stopped ART found a mean rate of decline of 2/year[50]. This is far higher than the mean rate of loss of 0.18/year found by a study of ART naïve individuals[49]. The plausible range for resistance transmission probabilities in ART dropouts was calculated from data on the rate of loss of detectable resistance mutations[49], and data on the rate of restarting ART after dropping out[32].

Figure 3. Plausible ranges for probability of transmitting a resistance mutation, given HIV transmission, by treatment status and CD4 count.

# Fitting methods

The model was fitted to 50 outputs using history matching with model emulation. A further output, the proportion of people who were switched to 2^nd^ line ART who were switched due to treatment failure resulting from drug resistance, was then fitted to by keeping the 100/200 model fits that gave the closest fit to the plausible range for the output.

# Cost data and analysis

Plausible ranges for 1^st^ and 2^nd^ line drug costs were based on minimum and maximum amounts paid per person year in Uganda in 2014 for recommended 1^st^ and 2^nd^ line drug regimens[30, 51]. The plausible range for the cost of providing pre-ART care and the 1^st^ six months of ART care (excluding drug costs) were based on estimates of the program costs of providing care at PEPFAR-supported outpatient clinics in Uganda[52]. As only a point estimate was given, and costs in Uganda were lower than costs in other countries, the plausible ranges were set to 50%-200% of the point estimates. The same study was used to calculate the plausible range for the reduction in program costs for people on established ART (continuously on ART for greater than six months). Four studies from Uganda were used to determine the plausible ranges for HIV[53, 54] and CD4[55, 56] test costs.

Pitter *et al* provided plausible ranges for costs in Uganda of clinic visits and hospitalisations (per night), for the average duration of hospitalisations[57]. Rates of hospitalisations by CD4 count for people not receiving ARVs or cotrimoxazole were available from a study in Uganda conducted before the widespread availability of ART or pre-ART care[16]. Logarithmic equations were fitted to the upper and lower 95% confidence intervals for rates of hospitalisations by CD4 count, and the equations were used to determine both the relationship between CD4 count and hospitalisations in the model, and the plausible range for a parameter which determined the overall frequency of hospitalisations at any CD4 count. The equation used was: hospital_‌nights_‌parameter * ln(CD4_count) - 6.44 * hospital_‌nights_‌parameter. Rates of hospitalisations at all CD4 counts were assumed to be lower for people in pre-ART care than for people not receiving care, and lower still for people on ART. Plausible ranges for the reductions in rates of hospitalisations at all CD4 counts based on data from the same study[16]. Finally, rates of clinic visits were assumed to be 2-5 times higher than rates of hospitalisations[58].

The plausible ranges for all cost parameters used in the analysis are given in Table 2. All costs are in 2015 USD, and costs were discounted at a rate of 3% per year.

## Costs of implementing interventions

In addition to the direct costs arising from more people being on ART or in pre-ART care, there will also be costs associated with implementing the interventions.

Increased rates of HIV testing will require interventions such as new testing locations (e.g. new fixed sites, or a door to door testing programme) and/or additional public health campaigns promoting testing. Studies in Uganga suggest that door-to-door testing programmes may be no more expensive than clinic-based testing[59, 60], however other interventions may increase the cost of HIV testing. On the other hand, HIV self-testing may be substantially cheaper than provider-delivered stategies[61]. In the increased HIV testing and test and treat interventions, we assume that any additional, intervention-related HIV tests (i.e. over and above the number in the baseline scenario in the same year) can be cheaper or more expensive than standard HIV tests. The plausible range for the change in cost was set to -50% to 50%.

Health system strengthening or other interventions will be required to increase the probability that someone who is diagnosed with HIV is linked to care. We assume that the cost of this will be proportional to the number of people who test positive for HIV. No suitable cost data were found to inform the costs of improving linkage to care. A wide plausible range of 0-20 USD was therefore chosen for the cost per positive HIV test.

Costs for improving pre-ART care, reducing ART dropout rates, and increasing ART restart rates were assumed to be proportional to the number of people in pre-ART care, the number of people on ART, and the number of people who have dropped out of ART respectively. Costs were based on a study in Rakai, Uganda which used peer health workers to improve retention in its ART programme, reducing loss to follow up by nearly 50%[62, 63]. The programme was estimated to cost 10-18 USD per person receiving ART per year. The study was conducted at an atypical location however - an ongoing reseach site - which may have affected costs. A wider plausible range of 0-50 USD per person per year was therefore used in this analysis. As no data were available to inform the costs of a program to increase restart rates, the same plausible range for per person costs was used for the improved ART restart rates intervention. Finally, as a similar community health worker based intervention could be used to improve pre-ART care, the same plausible range for per person costs was used for the improved pre-ART care intervention.

| Name | Description | Plausible range | Source |
| --- | --- | --- | --- |
| 1^st^_‌line_‌drug_‌cost | Annual cost of 1^st^ line antiretroviral drugs, per person | 118-137 | Uganda Ministry of Health (2013) [30] and WHO (2015) [51] |
| 2nd_‌line_‌drug_‌cost | Annual cost of 2^nd^ line antiretroviral drugs, per person | 151-330 | Uganda Ministry of Health (2013) [30] and WHO (2015) [51] |
| preART_‌program_‌cost | Annual pre-ART program costs, per person | 79-316 | Menzies *et al* (2011) [52] |
| early_‌ART_‌program_‌cost | Annual program costs of providing ART for 1^st^ six months, per person | 112-449 | Menzies *et al* (2011) [52] |
| reduced_‌cost_‌established_‌ART | Reduction in program costs after 6 continuous months on an ART regimen | 0.7-1 | Menzies *et al* (2011) [52] |
| HIV_‌test_‌cost | Cost per HIV test | 5.51-7.05 | Nichols *et al.* (2014) [53] and Mulogo *et al* (2013) [54] |
| CD4_‌test_‌cost | Cost per CD4 test | 5.18-17.48 | Kahn *et al* (2011) [55] and Lara *et al* (2012) [56] |
| clinic_‌visit_‌cost | Average cost per clinic visit (due to HIV-related morbidity) | 2.49-9.94 | Pitter *et al* (2007) [57] |
| hospital_‌night_‌cost | Average cost of a night’s stay in hospital | 3.95-15.80 | Pitter *et al* (2007) [57] |
| nights_‌per_‌hospital_‌visit | Average duration of an inpatient hospital stay, in nights | 3-7 | Pitter *et al* (2007) [57] |
| ‌hospital_‌nights_‌parameter | Determines the relationship between CD4 count and the rate of inpatient hospital stays per year for HIV+ people not receiving ART or pre-ART care. | -147.9 - -79.4 | Mermin *et al.* (2008) [16] |
| reduced_‌hospital_‌pre-ART_‌care | Reduction in inpatient hospital visits for HIV+ people receiving pre-ART care | 0.48-0.98 | Mermin *et al.* (2008) [16] |
| reduced_‌clinic_‌pre-ART_‌care | Reduction in clinic visits for HIV+ people receiving pre-ART care | 0.73-0.995 | Mermin *et al.* (2008) [16] |
| reduced_‌hospital_‌ART | Increased reduction in inpatient hospital visits for HIV+ people on ART compared to people receiving pre-ART care | 0.32-0.78 | Mermin *et al.* (2008) [16] |
| clinic_‌hospital_‌visit_‌ratio | Ratio of clinic visits to inpatient hospital stays | 2-5 | Mermin *et al* (2004) [58] |

**Table 2. Cost parameters used in the analysis of model results**

# Disability-adjusted life years

Four disability weight parameters were used in estimating the impact of interventions:

1. *Daly_slope_parameter.* This parameter determined the relationship between CD4 count and disability. Disability weight estimates and 95% confidence interval were available from the Global Burden of Disease Study 2010 for ‘HIV: symptomatic, pre-AIDS’, and ‘AIDS: not receiving antiretroviral treatment’[64]. In line with other modelling studies[65, 66], we assumed that these corresponded roughly to CD4 counts of 100 cells/μl and 275 cells/μl respectively, using the reported 95% confidence intervals as plausible ranges. To estimate disability weights at other CD4 counts, data on the rates of WHO stage 3/4 conditions by CD4 count[29] were used to estimate the shape of the relationship between CD4 count and morbidity at CD4 counts ≤350 cells/μl. At CD4 counts >350 cells/μl, disability weights were assumed to be equal to the weight at a CD4 count of 350 cells/μl[29]
2. *Daly_reduction_preART.* This parameter determined the relative reduction in disability weights while in pre-ART care (compared to an individual with the same CD4 count, who was not in HIV care). The plausible range was set equal to the 95% confidence interval of reductions in rates of hospitalisations after starting cotrimoxazole prophylaxis[16].
3. *Daly_ART.* The disability weight for people who had continuously been on ART for more than six months – ‘established ART’. The plausible range was set equal to the 95% confidence interval of the Global Burden of Disease Study’s estimate of disability for ‘HIV/AIDS: receiving antiretroviral treatment’[64].
4. *Daly_early_ART.* Determined the relative reduction in disability weights during the first six months on ART (compare to an individual with the same CD4 count in pre-ART care). The plausible range was set equal to the 95% confidence interval of reductions in mortality rates in the first 16 weeks after starting ART[16].

Bounds were placed on disability weights for people not in care, in pre-ART care, and in the 1^st^ six months on ART so they could not take values greater than the upper bound of the Global Burden of Disease Study’s 95% CI for disability weights for untreated AIDS, and could not take values lower than the disability weight for someone on established ART.

DALYs were discounted at a rate of 3% per year, and were not age-weighted.

| Name | Description | Plausible range | Source |
| --- | --- | --- | --- |
| *Daly_slope_parameter* | Parameter used to calculate disability weights by CD4 count for people not in HIV care. Weights are calculated using the equation *Daly_slope_parameter* * exp(-0.0071 * CD4_count) | 0.91-1.87 | Salomon 2012[64] and Anglaret 2012[29] |
| *Daly_reduction_preART* | Relative reduction in disability weights while in pre-ART care | 0.45-0.9 | Mermin 2008[16] |
| *Daly_ART* | Disability weight for people who had continuously been on ART for more than six months | 0.034-0.079 | Salomon 2012[64] |
| *Daly_early_ART* | Determined the relative reduction in disability weights during the first six months on ART | 0.27-0.74 | Mermin 2008[16] |

1. DeSA U: **World population prospects: The 2012 revision**. *Population Division of the Department of Economic and Social Affairs of the United Nations Secretariat, New York* 2013.

2. Kaleebu P, Kamali A, Seeley J, Elliott A, Katongole‐Mbidde E: **The Medical Research Council (UK)/Uganda Virus Research Institute Uganda Research Unit on AIDS–‘25 years of research through partnerships’**. *Tropical Medicine & International Health* 2014.

3. McCreesh N, O'Brien K, Nsubuga RN, Shafer LA, Bakker R, Seeley J, Hayes RJ, White RG: **Exploring the potential impact of a reduction in partnership concurrency on HIV incidence in rural Uganda: A modeling study**. *Sexually Transmitted Diseases* 2012, **39**(6):407-413 410.1097/OLQ.1090b1013e318254c318284a.

4. **AIDSInfo** [<http://www.unaids.org/en/dataanalysis/datatools/aidsinfo>]

5. Abaasa AM, Todd J, Ekoru K, Kalyango JN, Levin J, Odeke E, Karamagi CA: **Good adherence to HAART and improved survival in a community HIV/AIDS treatment and care programme: the experience of The AIDS Support Organization (TASO), Kampala, Uganda**. *BMC Health Services Research* 2008, **8**(1):241.

6. Muyingo SK, Walker AS, Reid A, Munderi P, Gibb DM, Ssali F, Levin J, Katabira E, Gilks C, Todd J: **Patterns of individual and population-level adherence to antiretroviral therapy and risk factors for poor adherence in the first year of the DART trial in Uganda and Zimbabwe**. *JAIDS Journal of Acquired Immune Deficiency Syndromes* 2008, **48**(4):468-475.

7. Byakika-Tusiime J, Crane J, Oyugi JH, Ragland K, Kawuma A, Musoke P, Bangsberg DR: **Longitudinal antiretroviral adherence in HIV+ Ugandan parents and their children initiating HAART in the MTCT-Plus family treatment model: role of depression in declining adherence over time**. *AIDS and Behavior* 2009, **13**(1):82-91.

8. Nachega JB, Hislop M, Dowdy DW, Lo M, Omer SB, Regensberg L, Chaisson RE, Maartens G: **Adherence to highly active antiretroviral therapy assessed by pharmacy claims predicts survival in HIV-infected South African adults**. *JAIDS Journal of Acquired Immune Deficiency Syndromes* 2006, **43**(1):78-84.

9. Kaleebu P, Ross A, Morgan D, Yirrell D, Oram J, Rutebemberwa A, Lyagoba F, Hamilton L, Biryahwaho B, Whitworth J: **Relationship between HIV-1 Env subtypes A and D and disease progression in a rural Ugandan cohort**. *Aids* 2001, **15**(3):293-299.

10. Kiwanuka N, Robb M, Laeyendecker O, Kigozi G, Wabwire-Mangen F, Makumbi FE, Nalugoda F, Kagaayi J, Eller M, Eller LA: **HIV-1 viral subtype differences in the rate of CD4+ T-cell decline among HIV seroincident antiretroviral naive persons in Rakai district, Uganda**. *Journal of acquired immune deficiency syndromes (1999)* 2010, **54**(2):180.

11. Ananworanich J, Gayet-Ageron A, Le Braz M, Prasithsirikul W, Chetchotisakd P, Kiertiburanakul S, Munsakul W, Raksakulkarn P, Tansuphasawasdikul S, Sirivichayakul S: **CD4-guided scheduled treatment interruptions compared with continuous therapy for patients infected with HIV-1: results of the Staccato randomised trial**. *The Lancet* 2006, **368**(9534):459-465.

12. DART Trial Team: **Fixed duration interruptions are inferior to continuous treatment in African adults starting therapy with CD4 cell counts< 200 cells/μl**. *Aids* 2008, **22**(2):237-247.

13. Staszewski S, Miller V, Sabin C, Schlecht C, Gute P, Stamm S, Leder T, Berger A, Weidemann E, Hill A: **Determinants of sustainable CD4 lymphocyte count increases in response to antiretroviral therapy**. *Aids* 1999, **13**(8):951-956.

14. El-Sadr W, Lundgren JD, Neaton J, Gordin F, Abrams D, Arduino R, Babiker A, Burman W, Clumeck N, Cohen C: **CD4+ count-guided interruption of antiretroviral treatment**. *New Engl J Med* 2006, **355**(22):2283-2296.

15. Bellan SE, Dushoff J, Galvani AP, Meyers LA: **Reassessment of HIV-1 acute phase infectivity: accounting for heterogeneity and study design with simulated cohorts**. *PloS Med* 2015, **12**(3):e1001801.

16. Mermin J, Were W, Ekwaru JP, Moore D, Downing R, Behumbiize P, Lule JR, Coutinho A, Tappero J, Bunnell R: **Mortality in HIV-infected Ugandan adults receiving antiretroviral treatment and survival of their HIV-uninfected children: a prospective cohort study**. *The Lancet* 2008, **371**(9614):752-759.

17. STD/AIDS Control Programme MoH: **Status of Antiretroviral Therapy Services in Uganda: Semi-Annual ART Report for January – June 2014**. In*.* Kampala, Uganda; 2014.

18. STD/AIDS Control Programme MoH: **Status of Antiretroviral Therapy Service Delivery in Uganda Quarterly Report for March – June 2010**. In*.* Kampala, Uganda; 2010.

19. Mills EJ, Bakanda C, Birungi J, Mwesigwa R, Chan K, Ford N, Hogg RS, Cooper C: **Mortality by baseline CD4 cell count among HIV patients initiating antiretroviral therapy: evidence from a large cohort in Uganda**. *Aids* 2011, **25**(6):851-855.

20. Todd J, Glynn JR, Marston M, Lutalo T, Biraro S, Mwita W, Suriyanon V, Rangsin R, Nelson KE, Sonnenberg P: **Time from HIV seroconversion to death: a collaborative analysis of eight studies in six low and middle-income countries before highly active antiretroviral therapy**. *Aids* 2007, **21**:S55-S63.

21. Uganda MoH, Macro O: **Uganda HIV/AIDS Sero-behavioural Survey 2004/2005**. In*.*: Ministry of Health Uganda and ORC Macro Kampala and Calverton; 2006.

22. Uganda Ministry of Health and ICF International: **2011 Uganda AIDS Indicator Survey: Key Findings**. In*.* Calverton, Maryland, USA: MOH and ICF International; 2012.

23. Suthar AB, Granich RM, Kato M, Nsanzimana S, Montaner JS, Williams BG: **Programmatic implications of acute and early HIV infection**. *Journal of Infectious Diseases* 2015:jiv430.

24. Hutchinson E, Parkhurst J, Phiri S, Gibb DM, Chishinga N, Droti B, Hoskins S: **National policy development for cotrimoxazole prophylaxis in Malawi, Uganda and Zambia: the relationship between Context, Evidence and Links**. *Health Research Policy and Systems* 2011, **9**(Suppl 1):S6.

25. Rosen S, Fox MP: **Retention in HIV care between testing and treatment in sub-Saharan Africa: a systematic review**. *PloS Med* 2011, **8**(7):e1001056.

26. Plazy M, Orne-Gliemann J, Dabis F, Dray-Spira R: **Retention in care prior to antiretroviral treatment eligibility in sub-Saharan Africa: a systematic review of the literature**. *BMJ Open* 2015, **5**(6).

27. STD/AIDS Control Programme MoH: **Status of Antiretroviral Therapy Service Delivery in Uganda Quarterly Report for October - December 2011**. In*.* Kampala, Uganda; 2011.

28. STD/AIDS Control Programme MoH: **Status of Antiretroviral Therapy Service Delivery in Uganda Quarterly Report for July-September 2013**. In*.* Kampala, Uganda; 2013.

29. Anglaret X, Minga A, Gabillard D, Ouassa T, Messou E, Morris B, Traore M, Coulibaly A, Freedberg KA, Lewden C: **AIDS and non-AIDS morbidity and mortality across the spectrum of CD4 cell counts in HIV-infected adults before starting antiretroviral therapy in Cote d’Ivoire**. *Clinical infectious diseases* 2012, **54**(5):714-723.

30. Uganda Ministry of Health: **Addendum To The Antiretroviral Treatment Guidelines For Uganda**. In*.* Kampala, Uganda; 2013.

31. STD/AIDS Control Programme MoH: **Status of Antiretroviral Therapy Service Delivery in Uganda Quarterly Report for July – September 2010**. In*.* Kampala, Uganda; 2010.

32. Kranzer K, Lewis JJ, Ford N, Zeinecker J, Orrell C, Lawn SD, Bekker L-G, Wood R: **Treatment interruption in a primary care antiretroviral therapy programme in South Africa: cohort analysis of trends and risk factors**. *Journal of acquired immune deficiency syndromes (1999)* 2010, **55**(3):e17.

33. Okero F, Aceng E, Madraa E, Namagala E, Serutoke J: **Scaling up antiretroviral therapy: Experience in uganda**. In*.* Geneva, Switzerland; 2003.

34. Shafer RW: **Rationale and Uses of a Public HIV Drug‐Resistance Database**. *Journal of Infectious Diseases* 2006, **194**(Supplement 1):S51-S58.

35. Rhee S-Y, Fessel WJ, Liu TF, Marlowe NM, Rowland CM, Rode RA, Vandamme A-M, Van Laethem K, Brun-Vezinet F, Calvez V: **Predictive value of HIV-1 genotypic resistance test interpretation algorithms**. *Journal of Infectious Diseases* 2009, **200**(3):453-463.

36. Zazzi M, Prosperi M, Vicenti I, Di Giambenedetto S, Callegaro A, Bruzzone B, Baldanti F, Gonnelli A, Boeri E, Paolini E: **Rules-based HIV-1 genotypic resistance interpretation systems predict 8 week and 24 week virological antiretroviral treatment outcome and benefit from drug potency weighting**. *Journal of antimicrobial chemotherapy* 2009, **64**(3):616-624.

37. Hamers RL, Schuurman R, Sigaloff KC, Wallis CL, Kityo C, Siwale M, Mandaliya K, Ive P, Botes ME, Wellington M: **Effect of pretreatment HIV-1 drug resistance on immunological, virological, and drug-resistance outcomes of first-line antiretroviral treatment in sub-Saharan Africa: a multicentre cohort study**. *The Lancet infectious diseases* 2012, **12**(4):307-317.

38. Stadeli KM, Richman DD: **Rates of emergence of HIV drug resistance in resource-limited settings: a systematic review**. *Antiviral therapy* 2013, **18**(1):115.

39. Gupta R, Hill A, Sawyer AW, Pillay D: **Emergence of drug resistance in HIV type 1-infected patients after receipt of first-line highly active antiretroviral therapy: a systematic review of clinical trials**. *Clinical infectious diseases* 2008, **47**(5):712-722.

40. Hoffmann CJ, Charalambous S, Sim J, Ledwaba J, Schwikkard G, Chaisson RE, Fielding KL, Churchyard GJ, Morris L, Grant AD: **Viremia, Resuppression, and Time to Resistance in Human Immunodeficiency Virus (HIV) Subtype C during First-Line Antiretroviral Therapy**. *Clinical Infectious Diseases* 2009, **49**(12):1928-1935.

41. El-Khatib Z, DeLong AK, Katzenstein D, Ekstrom AM, Ledwaba J, Mohapi L, Laher F, Petzold M, Morris L, Kantor R: **Drug resistance patterns and virus re-suppression among HIV-1 subtype C infected patients receiving non-nucleoside reverse transcriptase inhibitors in South Africa**. *Journal of AIDS & clinical research* 2011, **2**(117).

42. Von Wyl V, Yerly S, Böni J, Bürgisser P, Klimkait T, Battegay M, Furrer H, Telenti A, Hirschel B, Vernazza PL: **Emergence of HIV-1 drug resistance in previously untreated patients initiating combination antiretroviral treatment: a comparison of different regimen types**. *Archives of internal medicine* 2007, **167**(16):1782-1790.

43. Sigaloff KC, Hamers RL, Wallis CL, Kityo C, Siwale M, Ive P, Botes ME, Mandaliya K, Wellington M, Osibogun A: **Unnecessary antiretroviral treatment switches and accumulation of HIV resistance mutations; two arguments for viral load monitoring in Africa**. *JAIDS Journal of Acquired Immune Deficiency Syndromes* 2011, **58**(1):23-31.

44. Wawer MJ, Gray RH, Sewankambo NK, Serwadda D, Li X, Laeyendecker O, Kiwanuka N, Kigozi G, Kiddugavu M, Lutalo T *et al*: **Rates of HIV-1 transmission per coital act, by stage of HIV-1 infection, in Rakai, Uganda**. *J Infect Dis* 2005, **191**(9):1403-1409.

45. Nicolosi A, Leite MLC, Musicco M, Arid C, Gavazzeni G, Lazzarin A: **The Efficiency of Male-to Female and Female-to-Male Sexual Transmission of the Human Immunodeficiency Virus: A Study of 730 Stable Couples**. *Epidemiology* 1994, **5**(6):570-575.

46. Donnell D, Baeten JM, Kiarie J, Thomas KK, Stevens W, Cohen CR, McIntyre J, Lingappa JR, Celum C, Team PiPHHTS: **Heterosexual HIV-1 transmission after initiation of antiretroviral therapy: a prospective cohort analysis**. *The Lancet* 2010, **375**(9731):2092-2098.

47. Baggaley RF, White RG, Hollingsworth TD, Boily M-C: **Heterosexual HIV-1 infectiousness and antiretroviral use: systematic review of prospective studies of discordant couples**. *Epidemiology* 2013, **24**(1):110-121.

48. Jain V, Sucupira MC, Bacchetti P, Hartogensis W, Diaz RS, Kallas EG, Janini LM, Liegler T, Pilcher CD, Grant RM: **Differential persistence of transmitted HIV-1 drug resistance mutation classes**. *Journal of Infectious Diseases* 2011, **203**(8):1174-1181.

49. Castro H, Pillay D, Cane P, Asboe D, Cambiano V, Phillips A, Dunn DT, Aitken C, Webster D, Chadwick D: **Persistence of HIV-1 transmitted drug resistance mutations**. *Journal of Infectious Diseases* 2013, **208**(9):1459-1463.

50. Lawrence J, Hullsiek KH, Thackeray LM, Abrams DI, Crane LR, Mayers DL, Jones MC, Saldanha JM, Schmetter BS, Baxter JD: **Disadvantages of structured treatment interruption persist in patients with multidrug-resistant HIV-1: final results of the CPCRA 064 study**. *JAIDS Journal of Acquired Immune Deficiency Syndromes* 2006, **43**(2):169-178.

51. **Global Price Reporting Mechnism**

52. Menzies NA, Berruti AA, Berzon R, Filler S, Ferris R, Ellerbrock TV, Blandford JM: **The cost of providing comprehensive HIV treatment in PEPFAR-supported programs**. *AIDS (London, England)* 2011, **25**(14):1753.

53. Nichols BE, Sigaloff KC, Kityo C, Hamers RL, Baltussen R, Bertagnolio S, Jordan MR, Hallett TB, Boucher CA, de Wit TFR: **Increasing the use of second-line therapy is a cost-effective approach to prevent the spread of drug-resistant HIV: a mathematical modelling study**. *Journal of the International AIDS Society* 2014, **17**(1).

54. Mulogo E, Batwala V, Nuwaha F, Aden A, Baine O: **Cost effectiveness of facility and home based HIV voluntary counseling and testing strategies in rural Uganda**. *African health sciences* 2013, **13**(2):423-429.

55. Kahn JG, Marseille E, Moore D, Bunnell R, Were W, Degerman R, Tappero JW, Ekwaru P, Kaharuza F, Mermin J: **CD4 cell count and viral load monitoring in patients undergoing antiretroviral therapy in Uganda: cost effectiveness study**. *Bmj* 2011, **343**:d6884.

56. Lara AM, Kigozi J, Amurwon J, Muchabaiwa L, Wakaholi BN, Mota REM, Walker AS, Kasirye R, Ssali F, Reid A: **Cost effectiveness analysis of clinically driven versus routine laboratory monitoring of antiretroviral therapy in Uganda and Zimbabwe**. *PloS one* 2012, **7**(4).

57. Pitter C, Kahn JG, Marseille E, Lule JR, McFarland DA, Ekwaru JP, Bunnell R, Coutinho A, Mermin J: **Cost-effectiveness of cotrimoxazole prophylaxis among persons with HIV in Uganda**. *JAIDS Journal of Acquired Immune Deficiency Syndromes* 2007, **44**(3):336-343.

58. Mermin J, Lule J, Ekwaru JP, Malamba S, Downing R, Ransom R, Kaharuza F, Culver D, Kizito F, Bunnell R: **Effect of co-trimoxazole prophylaxis on morbidity, mortality, CD4-cell count, and viral load in HIV infection in rural Uganda**. *The Lancet* 2004, **364**(9443):1428-1434.

59. Menzies N, Abang B, Wanyenze R, Nuwaha F, Mugisha B, Coutinho A, Bunnell R, Mermin J, Blandford JM: **The costs and effectiveness of four HIV counseling and testing strategies in Uganda**. *Aids* 2009, **23**(3):395-401.

60. Tumwesigye E, Wana G, Kasasa S, Muganzi E, Nuwaha F: **High uptake of home-based, district-wide, HIV counseling and testing in Uganda**. *AIDS patient care and STDs* 2010, **24**(11):735-741.

61. Cambiano V, Mavedzenge SN, Phillips A: **Modelling the Potential Population Impact and Cost-Effectiveness of Self-Testing for HIV: Evaluation of Data Requirements**. *AIDS and Behavior* 2014, **18**(4):450-458.

62. Chang LW, Kagaayi J, Nakigozi G, Serwadda D, Quinn TC, Gray RH, Bollinger RC, Reynolds SJ, Holtgrave D: **Cost analyses of peer health worker and mHealth support interventions for improving AIDS care in Rakai, Uganda**. *AIDS care* 2013, **25**(5):652-656.

63. Chang LW, Kagaayi J, Nakigozi G, Ssempijja V, Packer AH, Serwadda D, Quinn TC, Gray RH, Bollinger RC, Reynolds SJ: **Effect of Peer Health Workers on AIDS Care in Rakai, Uganda: A Cluster-Randomized Trial**. *PLoS ONE* 2010, **5**(6):e10923.

64. Salomon JA, Vos T, Hogan DR, Gagnon M, Naghavi M, Mokdad A, Begum N, Shah R, Karyana M, Kosen S: **Common values in assessing health outcomes from disease and injury: disability weights measurement study for the Global Burden of Disease Study 2010**. *The Lancet* 2013, **380**(9859):2129-2143.

65. Martin NK, Devine A, Eaton JW, Miners A, Hallett TB, Foster GR, Dore GJ, Easterbrook PJ, Legood R, Vickerman P: **Modeling the impact of early antiretroviral therapy for adults coinfected with HIV and hepatitis B or C in South Africa**. *AIDS* 2014, **28**:S35-S46.

66. Eaton JW, Menzies NA, Stover J, Cambiano V, Chindelevitch L, Cori A, Hontelez JA, Humair S, Kerr CC, Klein DJ: **Health benefits, costs, and cost-effectiveness of earlier eligibility for adult antiretroviral therapy and expanded treatment coverage: a combined analysis of 12 mathematical models**. *The lancet global health* 2014, **2**(1):e23-e34.
